# Membrane connectivity estimated by digital image analysis of HER2 immunohistochemistry is concordant with visual scoring and fluorescence in situ hybridization results: algorithm evaluation on breast cancer tissue microarrays

**DOI:** 10.1186/1746-1596-6-87

**Published:** 2011-09-23

**Authors:** Aida Laurinaviciene, Darius Dasevicius, Valerijus Ostapenko, Sonata Jarmalaite, Juozas Lazutka, Arvydas Laurinavicius

**Affiliations:** 1 Institute of Oncology Vilnius University, Santariskiu 1, LT-08660 Vilnius, Lithuania; 2 National Center of Pathology, P.Baublio 5, LT-08406 Vilnius, Lithuania; 3 Faculty of Medicine, Vilnius University, M.K.Ciurlionio 21, LT-03101 Vilnius, Lithuania; 4 Faculty of Natural Sciences, Vilnius University, M.K.Ciurlionio 21, LT-03101 Vilnius, Lithuania

## Abstract

**Introduction:**

The human epidermal growth factor receptor 2 (HER2) is an established biomarker for management of patients with breast cancer. While conventional testing of HER2 protein expression is based on semi-quantitative visual scoring of the immunohistochemistry (IHC) result, efforts to reduce inter-observer variation and to produce continuous estimates of the IHC data are potentiated by digital image analysis technologies.

**Methods:**

HER2 IHC was performed on the tissue microarrays (TMAs) of 195 patients with an early ductal carcinoma of the breast. Digital images of the IHC slides were obtained by Aperio ScanScope GL Slide Scanner. Membrane connectivity algorithm (HER2-CONNECT™, Visiopharm) was used for digital image analysis (DA). A pathologist evaluated the images on the screen twice (visual evaluations: VE1 and VE2). HER2 fluorescence *in situ *hybridization (FISH) was performed on the corresponding sections of the TMAs. The agreement between the IHC HER2 scores, obtained by VE1, VE2, and DA was tested for individual TMA spots and patient's maximum TMA spot values (VE1max, VE2max, DAmax). The latter were compared with the FISH data. Correlation of the continuous variable of the membrane connectivity estimate with the FISH data was tested.

**Results:**

The pathologist intra-observer agreement (VE1 and VE2) on HER2 IHC score was almost perfect: kappa 0.91 (by spot) and 0.88 (by patient). The agreement between visual evaluation and digital image analysis was almost perfect at the spot level (kappa 0.86 and 0.87, with VE1 and VE2 respectively) and at the patient level (kappa 0.80 and 0.86, with VE1max and VE2max, respectively). The DA was more accurate than VE in detection of FISH-positive patients by recruiting 3 or 2 additional FISH-positive patients to the IHC score 2+ category from the IHC 0/1+ category by VE1max or VE2max, respectively. The DA continuous variable of the membrane connectivity correlated with the FISH data (HER2 and CEP17 copy numbers, and HER2/CEP17 ratio).

**Conclusion:**

HER2 IHC digital image analysis based on membrane connectivity estimate was in almost perfect agreement with the visual evaluation of the pathologist and more accurate in detection of HER2 FISH-positive patients. Most immediate benefit of integrating the DA algorithm into the routine pathology HER2 testing may be obtained by alerting/reassuring pathologists of potentially misinterpreted IHC 0/1+ versus 2+ cases.

## Introduction

Recent progress of virtual microscopy and digital image analysis technologies opens new perspectives for the development of more reliable tools of tissue-based biomarker measurement [1-4]. This would enable high-throughput research, quality assurance, and decision-support measures to control for observer variability. Not surprisingly, the dawn of digital pathology is marked by the efforts to optimise image analysis algorithms for HER2 expression in breast cancer tissue [4-7]. They all aim at ensuring accurate and reproducible measurement of HER2 expression, which correlates with pathologist's evaluation, amplification of the gene and clinical outcomes. In the absence of a true "gold standard", the objectivity of image analysis tools can also be tested by inter-algorithm variation studies [8]. Some studies have compared outputs of various tools for HER2 IHC analysis [9,10]. Computer-aided digital microscopy has been shown to reduce observer variability in HER2 IHC evaluation [11].

We designed our study to test the performance of HER2 IHC scoring based on a novel membrane connectivity estimate in tissue microarrays (TMAs) of breast cancer tissue. The digital analysis (DA) results were compared with the data of visual evaluation (VE) of HER2 by IHC and HER2 FISH test results on the same TMAs.

## Materials and methods

### Tumour Specimens

Tumour samples were obtained from prospectively collected series of 195 patients with an early invasive ductal carcinoma of the breast treated at the Oncology Institute of Vilnius University and investigated at the National Center of Pathology during the period of 2007 to 2009. The median age of the patients was 57 years (range 27-87 years). The patients were diagnosed with stage T1-2 tumours, without distant metastases (M0), however, 48% of the patients showed lymph node involvement (N1 or N2). Informed consent was obtained and documented in writing before study entry. The study was approved by the Lithuanian Bioethics Committee.

### Tissue Microarrays

The TMAs were constructed from 10% buffered formalin-fixed paraffin-embedded tissue blocks, selected by the pathologist (DD). The corresponding hematoxylin and eosin-stained slides were scanned by Aperio ScanScope GL Slide Scanner (Aperio Technologies, Vista, CA, USA) under 20 × magnification. The pathologist randomly selected and marked representative areas of the tumour on the whole section images. The images were then converted into Mirax MViewMRXS format and used to guide the production of the TMAs on the tissue arraying instrument (3DHISTECH, TMA Master, Budapest, Hungary). One millimetre-diameter cores were punched from the selected areas, thus producing 11 TMAs blocks containing 737 spots from 195 patients. Paraffin sections of the TMAs were cut for IHC (3 μm-thick) and FISH testing (4 μm-thick).

### Immunohistochemistry

The sections were immunostained on Ventana BenchMark XT staining system (Ventana Medical Systems, Tucson, Arizona, USA). Sections were deparaffinized in xylene, dehydrated through three alcohol changes and transferred to Ventana Wash solution. Epitope retrieval was performed on the slides using Cell Conditioning solution (pH 8.5) at 100°C for 36 min. The sections were then incubated with Ventana PATHWAY anti-HER2/neu (4B5) rabbit monoclonal antibody at 37°C for 16 min using Ventana Ultraview DAB detection kit. Finally, the sections were developed in DAB at 37°C for 8 min, counterstained with Mayer's hematoxylin and mounted. Whole tissue sections of HER2-positive breast tumour tissue were used as positive tissue controls, while negative controls were performed by omitting the application of primary antibody. Digital images were captured using the Aperio ScanScope GL Slide Scanner (Aperio Technologies, Vista, CA, USA) under 20 × magnification.

### Visual evaluation of HER2 IHC images

Visual evaluation of HER2 IHC score was performed by the pathologist (DD) twice (VE1, VE2) with an interval of 2 months, based on the review of the images of individual spots on the computer monitor (Acer AL2616W). The IHC results were scored according to the United States Food and Drug Administration (FDA) criteria approved for the 4B5 HER2 rabbit monoclonal antibody. Each spot was graded individually with 0, 1+, 2+ or 3+ HER2 score. For further analysis the score 0 and 1+ was merged into negative (0/1+) HER2 category. Based on the common adequacy criteria (tissue integrity, presence and amount of tumour tissue, staining artefacts), the pathologist encoded individual spots as inadequate. Similarly, spots containing ductal carcinoma in situ (DCIS), with or without invasive carcinoma, were excluded from further analysis.

### Digital analysis of HER2 IHC images

Digital analysis of the HER2 IHC TMAs was performed on the same images as the visual evaluation. By using the Arrayimager software module from Visiopharm (Hoersholm, Denmark), individual digital images of each spot were automatically extracted from the whole slide images of the 11 TMAs. For each spot, a region-of-interest (ROI) was fully automatically defined by the tissue detecting algorithm of the Visiomorph software module (Visiopharm, Hoersholm, Denmark). To secure against the potential effect on digital analysis of possibly artificial staining of the edge of the tissue spot, the ROI was designed to have a distance to the nearest edge of 100 pixels (approximately 25 μm). Automatic area control ensured exclusion of severely destroyed or missing spots from the study, since a tissue spot was only included, if its ROI area exceeded 37,000 μm^2^, corresponding to approximately 5% of the ROI of an intact spot with a diameter of 1 mm. The spots containing inadequate tumour sample or DCIS were excluded from the DA by means of visual evaluation.

**Figure 1:**
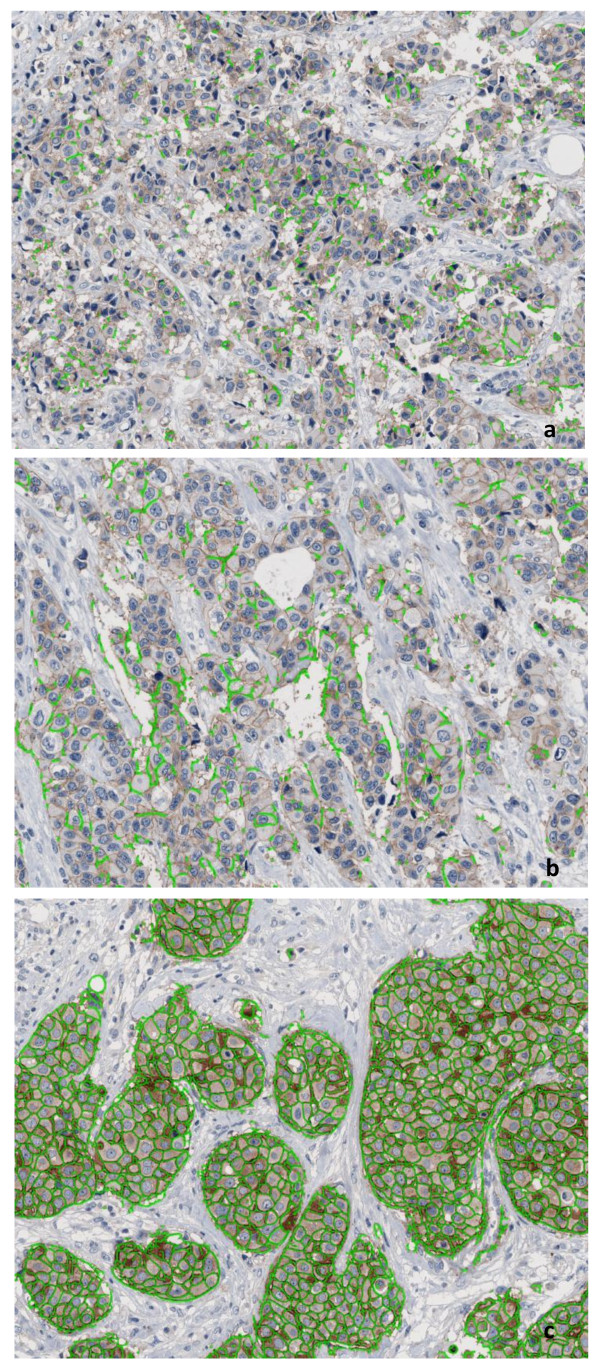
**Image outputs of the digital analyses**. Tissue microarray images scored by digital analysis as 0/1+ 2+ and 3+ (a. b. and c. respectively): green lines outline cell membranes revealing positive HER2 immunohistochemical staining by membrane connectivity estimate.

As recently described in detail [12], the DA was performed by the HER2-CONNECT™ software module (Visiopharm, Hoersholm, Denmark). Briefly, the algorithm of this software includes: 1) pre-processing for detection of pixels contributing to the characteristic brown linear structures in digital images of tissue sections immunostained for the presence of HER2 by the DAB substrate; 2) bimodal segmentation for distinguishing pixels representing stained membrane from all other pixels of the image; 3) post-processing for skeletonizing the membrane, merging membranes which were not perfectly connected, and eliminating small membrane fragments. The values of variable parameters used in the pre-processing, segmentation and post-processing were all established in a preceding study at NordiQC (Aalborg Hospital, Denmark) using different staining methods, another whole slide scanner, and manual outlining of ROI [12]. The parameters were not specifically optimized for the current study. The size of each membrane fragment is defined as the area of pixels its skeleton is composed of, and the connectivity is calculated from the size distribution of all membrane fragments within the ROI. The connectivity can vary continuously from 0, corresponding to a ROI without a single membrane fragment with an area larger than a pre-defined low cut-off, to 1, corresponding to a ROI for which all membrane fragments have areas larger than a pre-defined high cut-off. The continuous connectivity estimate was then converted into HER2 score: 0/1+ if connectivity ≤ 0.12, 2+ if 0.12 < connectivity ≤ 0.56, 3+ if 0.56 < connectivity ≤ 1, Figure 1.

### Fluorescence in situ hybridization

HER2 gene amplification was determined by a dual color FISH using the PathVysion HER2 DNA probe kit and Paraffin pretreatment kit (Abbott-Vysis, Inc., Downers Grove, IL, USA). Briefly, 4 μm sections were placed on positively charged slides and dried overnight at 56°C. The sections were deparaffinized in xylene, dehydrated in alcohol, air dried, then pretreated in 0.2N HCl for 20 min and in a pretreatment solution at 80°C for 30 min followed by protease digestion at 37°C for 26 min. Appropriate amount of hybridization solution containing directly labelled probes, both SpectrumGreen for the chromosome 17 centromere (CEP17) and SpectrumOrange for the HER2 gene locus, was applied, and the probe-target tissue was codenatured for 5 min at 72°C using Hybridizer (DAKO Diagnostics, Glostrup, Denmark) and allowed to hybridize for 19 h at 37°C. Non-hybridized probe was washed out in a hot 72°C 2 × SSC with 0.3% NP-40 solution for 2 min. Nuclei were counterstained with DAPI and coverslipped (Invitrogen Corporaton, Carlsbad, USA). Appropriate amplified and non-amplified in-house controls were processed in the run. Hybridized probes were examined manually by fluorescence Zeiss microscope (Zeiss, Axio Imager.Z2, Gottingen, Germany) equipped with a single green, orange and triple band pass filter Dapi-FITC-Cy3.

The FISH analyses for HER2 were performed manually without knowledge of the IHC result, according to Food and Drug Administration (FDA) scoring system in which HER2 gene amplification was set at an HER2/CEP17 ratio of more than 2. One evaluation per patient was performed after a review of a patient's spots in the TMAs and selection of a representative area in one of the spots for the FISH count (a total of 20 cells counted per patient).

### Statistical analysis

The agreement between VE1, VE2, and DA was tested by spot and by patient. The latter was based on the maximum HER2 score among the 2-4 spots belonging to the same patient (VE1max, VE2max, DAmax). The agreement was analyzed using kappa statistics; the strength of agreement 0.81-1.00 was interpreted as almost perfect [13]. The results are presented as weighted kappa with 95% confidence interval (CI). Pearson's correlation was performed to test the linear relationships between the continuous variable of membrane connectivity estimate and FISH results. Statistical analysis was performed with SAS 9.2 software.

## Results

### Sample (spot) adequacy

A total of 737 TMAs spots were evaluated visually by the pathologist twice (VE1 and VE2). After exclusion of spots containing inadequate samples or DCIS (n = 9), 575 spots remained for further analysis.

### Concordance of visual evaluation and digital analysis (by spot)

The pathologist intra-observer (VE1 *versus *VE2) agreement on HER2 score was almost perfect: kappa 0.91, 95% CI 0.88 - 0.95, Table 1. The percentage agreement was 96.0%. VE2 resulted in 4 spots shifting from 2+ to 3+ category and 4 spots from 3+ to 2+. Interestingly, VE2 "upgraded" 15 spots from 0/1+ to 2+ but no spots were "downgraded" from 2+ to 0/1+.

**Table 1:** Concordance of HER2 immunohistochemistry visual evaluation and digital analysis by each tissue microarray spot

**VE1 HER2 score**	**VE2 HER2 score**			
	**0/1+**	**2+**	**3+**	**Total**
	
0/1+	475	15	0	490
2+	0	21	4	25
3+	0	4	56	60
**VE1 HER2 score**	**DA HER2 score**			
	0/1+	2+	3+	Total
	
0/1+	458	32	0	490
2+	4	18	3	25
3+	0	0	60	60
**VE2 HER2 score**	**DA HER2 score**			
	0/1+	2+	3+	Total
	
0/1+	454	21	0	475
2+	8	26	6	40
3+	0	3	57	60

The concordance *per spot *is based on individual TMA spot HER2 score value: VE1 and VE2 - first and second visual evaluation by the pathologist; DA - digital analysis score.

Agreement between the VE and DA was almost perfect: VE1 versus DA, kappa - 0.86, 95% Cl: 0.81 - 0.90, VE2 versus DA, kappa - 0.87, 95% Cl: 0.82 - 0.91 (Table 1). The corresponding percentage agreement was 93.2% and 93.4%. In both analyses great majority of HER2 negative (0/1+) and positive (3+) spots by VE1 and VE2 were classified as such by the DA. Most of the discordance was present in the 2+ category where 72% and 65% of the 2+ spots by VE1 and VE2, respectively, were scored 2+ by the DA. On the other hand, 32 and 21 (6.5 and 4.4%) spots were recruited by the DA into 2+ category from the 0/1+ category by VE1 and VE2, respectively. Remarkably, no spots were discrepant in the interval of two categories (0/1+→3+ or 3+→0/1+).

### Concordance of visual evaluation and digital analysis (by patient)

To test the concordance of VE and DA score on a patient level, the cases with 2, 3 or 4 adequate spots (by both VE and DA) per patient were selected. Out of the 177 cases with a total of 575 adequate spots, 16, 15, 55, and 91 cases contained 1, 2, 3, and 4 adequate spots, respectively, thus leaving 161 patients with 2, 3 or 4 spots for further analysis.

Patient's IHC HER2 score was defined as maximum score (VE1max, VE2max, DAmax) obtained from the 2-4 spots analyzed. Remarkably, variation of the HER2 score between patient's spots was rather low: all (2, 3 or 4) spots revealed the same score in 156, 151, and 141 patients evaluated by VE1, VE2, and DA, respectively (Table 2). Thus, in a great majority of the 161 patients, the individual spots produced the same result per patient which would be identically expressed as maximum, mode, or median. The remaining 4, 10, and 19 patients (VE1, VE2, and DA, respectively) revealed a range of 1; remarkably, the great majority of this variation was observed for the 2+ category. Only one patient revealed the "inter-spot" range of 2 by VE1 (3 spots graded 0/1+, 2+, 3+ *versus *2+, 2+, 3+ by both VE2 and DA) with FISH ratio of 2.15 (potential false-negative if only the VE1 performed only on the first spot). Another patient revealed an "inter-spot" range of 2 by DA (4 spots graded 3+, 1+, 1+, 1+ *versus *2+, 2+, 2+, 1+ and 2+, 2+, 2+, 2+ by VE1 and VE2, respectively) with FISH ratio of 1.47 (potential false-positive if only the DA performed only on the first spot and/or DAmax used as the patient's HER2 score).

**Table 2:** Patients' HER2 immunohistochemistry score in the TMAs represented by a range and maximum spot value

**VE1range**	**VE1max**			
	**0/1+**	**2+**	**3+**	**Total**
	
0	136	4	16	156
1	0*	4	0	4
2	0*	0*	1	1
	
VE2range	VE2max			
	0/1+	2+	3+	Total
	
0	129	6	16	151
1	0*	9	1	10
2	0	0*	0	0
	
DArange	DAmax			
	1	2	3	Total
	
0	122	3	16	141
1	0*	16	3	19
2	0*	0*	1	1

* the range cannot logically be 1 or 2.

The cross tabulation of the patients' HER2 score range and maximum values represents variation of the analysis results between individual patient's spots. VE1max and VE2max - first and second visual evaluation by the pathologist, DAmax - digital analysis score. Accordingly, VE1range, VE2range, and DArange represent range between a minimum and maximum spot HER2 score value.

The pathologist intra-observer (VE1max *versus *VE2max) agreement on HER2 score was almost perfect: kappa 0.88, 95% CI0.81-0.96 (Table 3). The percentage agreement was 94.4%. By the VE2max, the number of 2+ patients increased from 8 to 15 due to 7 and 1 patients moving from VE1 0/1+ and 3+ categories, respectively.

**Table 3:** Concordance of HER2 immunohistochemistry maximum TMA score by visual evaluation and digital analysis

	VE2max HER2 score			
**VE1max HER2 score**	0/1+	2+	3+	Total
0/1+	129	7	0	136
2+	0	7	1	8
3+	0	1	16	17
	**DAmax HER2 score**			
	
**VE1max HER2 score**	0/1+	2+	3+	Total
0/1+	122	14	0	136
2+	0	5	3	8
3+	0	0	17	17
	**DAmax HER2 score**			
	
**VE2max HER2 score**	0/1+	2+	3+	Total
0/1+	121	8	0	129
2+	1	11	3	15
3+	0	0	17	17

The concordance *per patient *is based on maximum TMA spot HER2 score value: VE1max and VE2max - first and second visual evaluation by the pathologist, DAmax - digital analysis score.

The agreement between the visual and digital evaluation was almost perfect: VE1max versus DAmax, kappa 0.80, 95% CI 0.70-0.89; the VE2max versus DAmax, kappa 0.86, 95% CI 0.79-0.94 (Table 3). The percentage agreement was 89.4% and 92.5%, respectively. In all three analyses, 17 patients remained in the 3+ category. Similarly, 122 (80%) and 121 (94%) of HER2 negative (0/1+) patients by the VE1max and VE2max, respectively, were classified as such by the DA. Again, most of the discrepancies were present in the 2+ category where 62-73% of the 2+ patients by the VEmax were classified by DAmax as 2+. In general, the DAmax tended to "upgrade" HER2 score in some patients, shifting them from 0/1+ and 2+ categories into 2+ and 3+, accordingly. Remarkably, no discrepancies between the VEmax and DAmax in the interval of two categories were detected.

### Proportion of HER2 FISH-positive cases in the IHC categories scored by the visual evaluation and digital analysis

HER2 FISH test was performed on the sections from the same TMAs containing the 575 spots used for the IHC analysis. FISH results of overall 152 patients were obtained and compared with the HER2 IHC results (Table 4). The raw data of the patients with IHC score 2+ or 3+ (by either visual evaluation or digital analysis) and/or FISH HER2/CEP17 ratio > 2.0 and/or CEP17 > 3.0 is presented in the Table 5.

**Table 4:** Proportion of HER2 FISH-positive cases in the IHC score categories by visual evaluation and digital analysis

	HER2 FISH positive (%)		
	
HER2 score	VE1max	VE2max	DAmax
0/+1	8/127 (6.3)	7/120 (5.8)	5/113 (4.4)
2+	2/8 (25.0)	2/15 (13.3)	3/19 (15.8)
3+	14/17 (82.4)	15/17 (88.2)	16/20 (80.0)
Total	152	152	152

Number and percentage (in parentheses) of FISH-positive patients in the IHC score categories by maximum TMA spot HER2 score value: VE1max and VE2max - first and second visual evaluation by the pathologist, DAmax - digital analysis score.

**Table 5:** Raw data of the FISH, visual evaluation and digital analysis results

Line #	Her2/CEP17	HER2	CEP17	VE1max	VE2max	DAmax
1	0,4	1,8	4,2	1	1	1
2	0,5	2,1	4,4	1	1	1
3	0,5	1,9	3,5	1	1	1
4	0,9	3,0	3,4	1	1	1
5	1,1	3,7	3,5	1	1	1
6	1,3	4,2	3,3	1	1	1
7	1,7	5,4	3,1	1	1	1
8	1,8	5,7	3,2	1	1	1
9	1,8	7,1	3,9	1	1	1
10	2,1	2,7	1,3	1	1	1
11	2,3	4,2	1,9	1	1	1
12	2,4	3,2	1,3	1	1	1
13	2,7	3,8	1,4	1	1	1
14	4,6	12,6	2,8	1	1	1
15	1,2	1,8	1,6	1	1	2
16	1,3	2,3	1,8	1	1	2
17	1,4	5,7	3,9	1	1	2
18	1,5	6,7	4,5	1	1	2
19	1,6	4,3	2,7	1	1	2
20	1,6	5,3	3,3	1	1	2
21	2,1	3,8	1,8	1	1	2
22	4,5	4,5	1,0	1	1	2
23	0,5	1,5	2,9	1	2	1
24	1,0	2,1	2,1	1	2	2
25	1,3	4,7	3,7	1	2	2
26	1,4	4,1	2,8	1	2	2
27	1,7	5,8	3,5	1	2	2
28	2,0	5,4	2,8	1	2	2
29	2,2	4,3	1,9	1	2	2
30	1,0	4,9	4,7	2	2	2
31	1,1	4,1	3,7	2	2	2
32	1,2	3,0	2,4	2	2	2
33	1,3	5,0	3,8	2	2	2
34	1,4	3,3	2,4	2	2	2
35	1,5	3,3	2,2	2	2	3
36	3,4	11,3	3,4	2	2	3
37	6,5	12,4	1,9	2	3	3
38	1,7	9,0	5,2	3	2	3
39	1,4	2,6	1,8	3	3	3
40	1,5	4,6	3,1	3	3	3
41	2,3	4,3	1,9	3	3	3
42	4,5	4,5	1,0	3	3	3
43	4,7	10,1	2,2	3	3	3
44	4,9	11,2	2,3	3	3	3
45	4,9	15,5	3,2	3	3	3
46	5,4	13,8	2,6	3	3	3
47	5,9	16,8	2,9	3	3	3
48	6,5	20,4	3,2	3	3	3
49	6,8	13,6	2,0	3	3	3
50	7,2	22,7	3,2	3	3	3
51	7,2	23,2	3,2	3	3	3
52	8,3	22,0	2,7	3	3	3
53	13,1	28,2	2,2	3	3	3
54	13,3	38,5	2,9	3	3	3

The raw data of the patients with IHC score 2+ or 3+ (by either visual evaluation or digital analysis) and/or FISH HER2/CEP17 ratio > 2.0 and/or CEP17 > 3.0

The same 5 FISH-positive patients were present in the merged category of IHC 0 or 1+, established by all evaluations (VE1max, VE2max, and DAmax). The HER2/CEP17 ratio was in the range of 2.1 to 2.7 in 4 patients, one patient revealing HER2 gene clusters with HER2/CEP17 ratio of 4.6 (Table 5, lines 10-14). These 5 cases were not likely to be false-negative by IHC since their initial (diagnostic; data not shown) IHC result was negative and no issues with formalin fixation of the samples were noted. In the category 0/1+ (Table 4), VE2max, and VE1max included additional (2 and 3, respectively) FISH-positive patients with the HER2/CEP17 ratio of 2.1, 2.2, and 4.5 (Table 5, lines 21, 22, 29; potential false-negatives if only the visual evaluations were performed).

In the categories of IHC 2+ and 3+ (Table 4), most (3+16 = 19) FISH-positive patients were detected by DAmax, followed VE2max (2+15 = 17), and VE1max (2+14 = 16). On the other hand, DAmax 3+ picked up 4 patients with the HER2/CEP17 ratio 1.4-1.7 (Table 5, lines 35, 38-40), however, 1 of these patients (line 38) revealed the gene amplification along with polysomy (mean HER2 = 9.0; CEP17 = 5.2). The same 4 patients fell into either 3+ or 2+ category by VE1max and VE2max.

In summary, DAmax appeared to be most accurate with respect to positive FISH results. For the most of the cases where discrepancy was observed between IHC and FISH, the VE and DA were in agreement, and the discrepancy therefore seemed to be related to either biological variation of HER2 amplification and expression, or due to mistakes in reagents or assay procedures.

### Correlation of membrane connectivity estimate with HER2 FISH results

**Figure 2:**
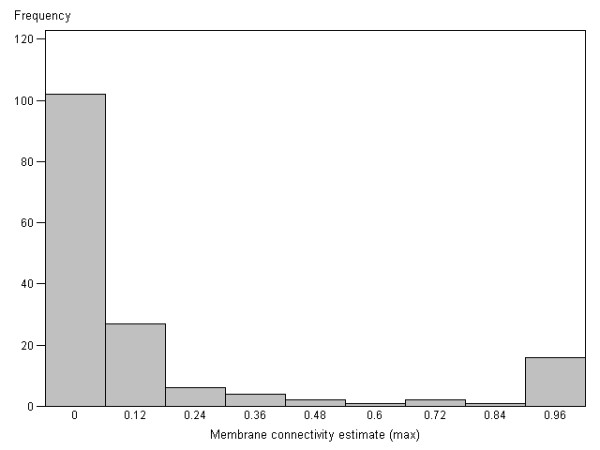
**Histogram of distribution of the patients by their maximum TMA spot HER2 membrane connectivity estimate**. Histogram plot represents distribution of the patients by their maximum spot HER2 membrane connectivity estimate.

Digital analysis of the IHC HER2 is based on the continuous variable of membrane connectivity and can be used in analyses of biomarker expression, independently of categorical scoring systems. We explored the potential of the membrane connectivity estimate comparing it to the patient's HER2 FISH data. Maximum spot value (ConnectMax) was used to characterize the patient's membrane connectivity estimate. Distribution analysis of the ConnectMax revealed pronounced bimodal pattern with left asymmetry (Figure 2). Significant correlations between log(ConnectMax) and the FISH results were observed: log(mean HER2) copy number per cell (r = 0.67, p < 0.0001), log(mean HER2/CEP17) ratio (r = 0.57, p < 0.0001), and mean CEP17 number per cell (r = 0.39, p < 0.0001).

**Figure 3:**
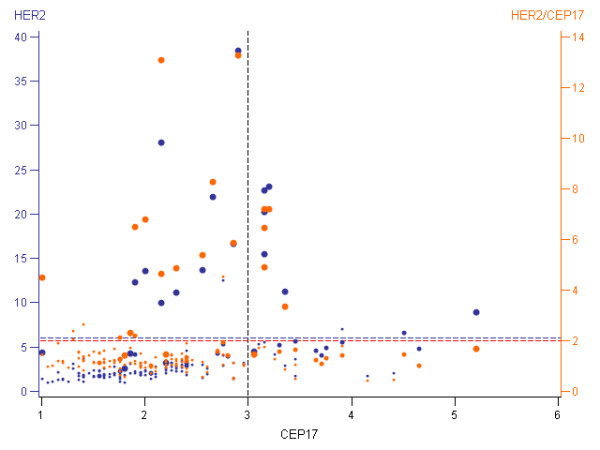
**Bubble plot display of interrelationship between the HER2 membrane connectivity estimate and HER2 FISH data**. Horizontal axis represents mean CEP17 by FISH analysis. Left vertical axis represents mean HER2 by FISH analysis. Right vertical axis represents HER2/CEP17 ratio. Bubble size is proportional to the patient's maximum HER2 connectivity value. Black dashed vertical reference line separates cases with polysomy (CEP17 > 3) to the right. Blue and orange dashed horizontal reference lines separate cases with amplification (HER2 > 6 and HER2/CEP17 > 2) above. Each patient is represented by two bubbles with the same CEP17 value: blue bubble maps to the mean HER2 orange bubble - to the mean HER2/CEP17 ratio.

These interrelationships with absolute and relative FISH variables raise an issue of understanding the complexity of the phenomena depicted in the bubble plot (Figure 3). Most IHC- and FISH-negative cases are represented by small dots in the left lower quadrant, while the positive cases concentrate in the left upper quadrant. However, quite numerous IHC-negative and IHC-positive cases fall into the "polysomy" quandrants on the right. The few IHC-FISH discrepancies can be tracked on the diagram, some of them revealing examples where conventional criteria for HER2 gene amplification testing by FISH may not always work. In particular, note the IHC-positive case with a high polysomy and mean HER2 per cell above 6, but the HER2/CEP17 ratio below 2 (also, Table 5, line 38). Multivariate analysis of the IHC and FISH parameters may help understanding these complexities, and the membrane connectivity estimate may serve as a continuous variable of the IHC positivity.

## Discussion

Our experiment revealed a reliable performance of HER2 expression measurement by the IHC digital image analysis based on the membrane connectivity estimate. The algorithm was run "plug-and-play" on the TMA images without an attempt to calibrate for potential image variation caused by scanning or IHC procedures. Manual annotation of the tumour tissue was not performed; however, spots containing DCIS or insufficient amount of tumour tissue were excluded from digital analysis by visual evaluation. Under these conditions, the digital analysis was in almost perfect agreement with the pathologist's score (VE) and exceeded the latter in terms of detecting FISH-positive patients.

We tested the agreement between the visual and digital evaluations in two sets of analyses: it was almost perfect at the level of individual spot (kappa 0.86 and 0.87, with the VE1 and VE2 respectively) and at the patient level (kappa 0.80 and 0.86, with the VE1max and VE2max, respectively). In general, the level of agreement in our study was among the highest reported when compared to that of previous studies using various digital analysis platforms [5,6,9,10,14-17], but obviously some caution has to be taken when comparing across studies with different designs. In both VE and DA, we used maximum TMA spot values to define patient's HER2 IHC status. This approach has been tested previously [18], and, in our view, is a better way to summarize TMA data per patient than mean or median value, especially, when tissue heterogeneity is a concern. Also, maximum spot value increases the sensitivity of HER2 detection and may compensate for the limited tissue sampling in TMA.

As expected from the previous studies [6,14,15], both 0/1+ and 3+ IHC categories were consistently discriminated by both the VE and DA, whereas most discrepancies were present in detection of the 2+ score category. Although it sounds like a paradox, these discrepancies may bring the greatest "added value" of integrating digital analysis into the routine pathology work-up of HER2 testing. Extrapolation of our experiment to clinical setting would mean that in the cohort of 152 patients with early ductal carcinoma of the breast, HER2 IHC evaluated by one pathologist once (VE1max) would have revealed 8 patients with HER2 IHC 2+ with 8 reflex FISH tests performed. Including the DA would have resulted in additional 14 HER2 IHC 2+ cases followed by the obligatory 14 FISH tests, thus detecting another 3 HER2-amplified cases (Table 5, lines 21, 22, 29). If the decision to perform a reflex FISH test were based on the IHC 2+ score by either VE1max or DA, that would have resulted in 19 FISH-positive cases compared to 16 by the VE1max-based decision alone (leading to 19% increase of the number of HER2-amplified cases in the cohort). In the setting where the pathologist would evaluate the IHC twice (VE1max and VE2max), the second review would have resulted in additional 8 HER2 IHC 2+ cases followed by the obligatory 8 FISH tests, thus detecting 1 additional HER2-amplified case; inclusion of the DA results into the account would require another 8 FISH tests with another 2 HER2-amplified cases detected. Considering potential consequences of a misdiagnosed HER2 status in 2 or 3 patients in the cohort of 152 for the "price" of adding automated digital analysis step and roughly 5-8 additional FISH tests per misdiagnosed case, the "balance" seems to be on the positive side. On the other hand, addition of the DA would have "saved" 2 or 3 FISH tests (compared to VE2max and VE1max, respectively) by suggesting the IHC 3+ score instead of the pathologist's 2+ score (Table 5, lines #35-37), however, one of the cases (#35) was negative by FISH, revealing potential lack of specificity of the DA alone. In contrast to other studies [19,20], our DA did not give a promise of a decreased number of IHC 2+ cases or increased specificity in detecting HER2-amplified cases. This latter statement, however, must be taken with caution since individual "sensitivity" of the pathologists may shift the VE results in different directions relative to the DA (the inter-observer variability was not tested in the present study). In summary, we suggest that the membrane connectivity DA would be most useful as a decision-support and quality assurance tool, alerting pathologists of borderline 0/1+ versus 2+ and 2+ versus 3+ HER2 IHC cases, thus improving the accuracy of the HER2 testing, but without expectation of significant savings by avoiding unnecessary FISH tests. Nevertheless, improved accuracy of the HER2 testing, without having to perform FISH in all cases, presents a reasonable economic trade-off. Although these considerations are based on the TMA analyses, whereas current pathology HER2 testing routine is based in the whole section samples, our data is at least representative and simulates the cases when limited tumour samples are available for testing.

The pathologist intra-observer agreement was slightly better than that with the digital analysis. However, the DA appeared to be more accurate in detection of FISH-positive patients. Interestingly, the second visual evaluation (VE2) was slightly more "sensitive" than VE1: it detected more 2+ patients and rescued 1 FISH-positive patient from the 0/1+ category by VE1. It is likely that this increase of sensitivity is a result of a learning curve - the pathologist adapting to evaluation of small samples of tissue in the TMAs as opposed to the IHC whole section slides used in routine pathology practice. This aspect may present additional benefit of the DA not only in the TMA analyses but also when a small tumour sample is available.

Objectivity of the digital analysis depends on numerous factors [8]; one particular factor is the accuracy of tumour tissue sampling for the analysis. If non-tumour tissue is included in the analysis, it may "dilute" the percentage of positive cells. In our experiment, no manual or automated annotation of the tumour tissue was performed, nevertheless, the DA recruited more 2+ and 3+ spots and patients than VE. Inevitably, our TMA spots contained variable proportions of tumour and non-tumour tissues and the digital analysis results could have been distorted without proper selection of the tumour tissue. However, since the membrane connectivity is a non-cell-based estimate and does not require distinction between tumour and non-tumour cells, the only prerequisite for the digital analysis was a sufficient amount but not proportion of tumour tissue in the ROI. This also provided the benefit of avoiding manual annotation of the ROI - the laborious and potentially biasing step of the image analysis.

With regard to detection of FISH-positive patients, the digital analysis provided maximum accuracy of IHC interpretation possible in our TMAs. As outlined in the Results section, the "false-positive" and "false-negative" cases by DAmax were also discrepant by VE1max and VE2max and most likely represented a true biological variation of HER2 gene amplification and expression and/or possible issues in tissue processing [21-26]. Although HER2 FISH status is commonly used as a "gold standard" in HER2 IHC studies, in a small proportion of cases it may remain discrepant due to tissue heterogeneity, CEP17 polysomy/amplification (if only HER2/CEP17 ratio is used to define the HER2 status), or other unrecognized causes of variation [27-30]. Our data reveal a subpopulation of patients where conventional HER2 FISH positivity criteria based on HER2/CEP17 ratio may be not sufficient and support the need to further explore the biological continuum of HER2 positivity and clinical relevance of the test [30-33]. Although analysis of this complexity is beyond the scope of the present study, it is important to note that the membrane connectivity estimate represents a continuous variable of HER2 expression by IHC and can serve better than categorical IHC score in statistical analyses exploring the relationships of HER2 expression and amplification. In support of this perspective, we found significant correlations of the IHC membrane connectivity with the FISH results: HER2 copy number (r = 0.67), HER2/CEP17 ratio (r = 0.57), and mean CEP17 number per cell (r = 0.39), similar to the recent report of Vranek et al [34] (although the correlation to CEP17 did not reach statistical significance in this study of patients with the CEP17 polysomy). Of note, automation and further quantification of the FISH testing, with increase of accuracy and capacity of the test, seems to be an important step to further progress.

## Conclusions

In conclusion, HER2 IHC digital image analysis based on membrane connectivity estimate, tested on early ductal carcinoma of the breast tissue microarrays, was in almost perfect agreement with the visual evaluation of the pathologist and more accurate in detection of HER2 FISH-positive patients. Most immediate benefit of integrating the DA algorithm into the routine pathology HER2 testing may be obtained by alerting/reassuring pathologists of potentially misinterpreted IHC 0/1+ versus 2+ cases. The algorithm was used without manual or automated annotation of tumour tissue and appeared to be independent of the proportion of tumour in the tissue analyzed. It provided a continuous variable reflecting HER2 IHC expression and could be useful for quality assurance, computer-assisted diagnosis, and HER2 amplification/expression heterogeneity studies.

## List of abbreviations

ConnectMax: patient's membrane connectivity estimate based on the maximum TMA spot value; DA: digital image analysis; DAB: diaminobenzidine; DCIS: ductal carcinoma in situ; FDA: Food and Drug Administration; FISH: fluorescence *in situ *hybridization; HER2: the human epidermal growth factor receptor 2; IHC: immunohistochemistry; ROI: region-of-interest; TMA/TMAs: Tissue microarray/Tissue microarrays; VE: visual evaluation.

## Competing interests

The authors declare that they have no competing interests.

## Authors' contributions

AiL and ArL contributed equally to the study. AiL designed and carried out the TMAs analyses, and drafted the manuscript. ArL conceived the study, participated in study design, performed statistical analysis, and edited the manuscript. DD performed visual evaluation of the TMAs images. VO, SJ, and JL participated in conception and design of the study, reviewing the analysis results and editing the manuscript. All authors read and approved the final manuscript.
